# Automated Negotiation for Resource Assignment in Wireless Surveillance Sensor Networks

**DOI:** 10.3390/s151129547

**Published:** 2015-11-24

**Authors:** Enrique de la Hoz, Jose Manuel Gimenez-Guzman, Ivan Marsa-Maestre, David Orden

**Affiliations:** 1 Departamento de Automática, Universidad de Alcalá, Edificio Politécnico, 28805 Alcalá de Henares (Madrid), Spain; E-Mails: enrique.delahoz@uah.es (E.H.); josem.gimenez@uah.es (J.M.G.-G.); 2 Departamento de Física y Matemáticas, Universidad de Alcalá, Edificio Politécnico, 28805 Alcalá de Henares (Madrid), Spain; E-Mail: david.orden@uah.es

**Keywords:** wireless sensor networks, surveillance, resource assignment, graphs, automated negotiation

## Abstract

Due to the low cost of CMOS IP-based cameras, wireless surveillance sensor networks have emerged as a new application of sensor networks able to monitor public or private areas or even country borders. Since these networks are bandwidth intensive and the radioelectric spectrum is limited, especially in unlicensed bands, it is mandatory to assign frequency channels in a smart manner. In this work, we propose the application of automated negotiation techniques for frequency assignment. Results show that these techniques are very suitable for the problem, being able to obtain the best solutions among the techniques with which we have compared them.

## 1. Introduction

Wireless multimedia sensor networks are a special type of wireless sensor networks that are able to manage multimedia content. According to [1], the development of these networks fosters the evolution of new applications, such as electronic surveillance, traffic monitoring and guidance, healthcare, assistance for elderly people and environmental monitoring. The research community has studied a wide range of aspects of these type of networks, as summarized in the surveys [1,2,3,4].

Security in residential and public areas is a matter of great importance for many people. For that reason, some Internet providers are offering surveillance systems as a value-added service. As many other fields in technology, surveillance systems have recently undergone a revolution, shifting from CCTVs to IP-based cameras, as the latter offer clear advantages to their users. Not only their cost is more competitive, but IP-based cameras are also able to naturally connect to the widely-deployed Internet. Thus, IP-based cameras will constitute the key component in surveillance systems that are connected to the Internet, setting up a wireless surveillance sensor network (WSSN).

The main requirements of a WSSN combine the requirements from wireless sensor networks and real-time applications, the high bandwidth demand and low power consumption standing out. The network must provide for surveillance purposes a high bandwidth, as video quality must be high enough to distinguish even patterns that may indicate intrusion or attack attempts. The low power consumption constraint comes from the essence of wireless sensor networks, as sensors must be as autonomous as possible.

WSSNs make use of radio resources in unlicensed frequency bands, which currently are highly populated with a wide range of devices using heterogeneous technologies. Moreover, the requirements in terms of bandwidth are also increasing. All of these motives as a whole make it harder for wireless communication networks to meet user demands regarding quality of service. For all of these reasons, we can claim that there is an actual need for a more efficient management of the radio spectrum. Many solutions have been proposed for the problem of radio spectrum scarcity from different points of view. Some works focus on improving spectral efficiency [5] and error detection and correction algorithms [6] or on the design of new antennas for taking better advantage from multipath propagation for a more efficient signal reception (MIMO) [7]. Some other works propose, for a certain wireless technology, device coordination mechanisms for those devices that share the same frequency band, so that interferences are minimized and throughput is increased. For data services, the effect of interferences on the throughput perceived by the user is of paramount significance, as throughput ranges from zero until its maximum value depending on the SINR (signal-to-interference plus noise ratio; determines the relation between the power of the desired signal (the video we want to receive) and all of the undesired signals that are received (other video flows and noise)) value [8].

This last problem is commonly known as the frequency assignment problem (FAP) [9]. Frequency assignment has been approached in a centralized manner from the point of view of optimization [10] and from an engineering point of view with distributed heuristics, LCCS (least congested channel search) [11] being one of the most commonly-used techniques.

Therefore, to deploy the required bandwidth to IP-based cameras in a WSSN environment, frequency assignment arises as a paramount problem. In this work, we address the frequency (channel) assignment problem in WSSNs from the perspective of automated negotiation techniques. Automated negotiation has proven to be valuable to support the decision-making process in scenarios where it is necessary to find an agreement quickly and with conflicting interests involved [12]. Potential applications of automated negotiation range from e-commerce [13] to task distribution problem solving, resource sharing or cooperative design [14,15,16,17]. One of the most important advantages of automated negotiation is that it takes into account the conflict of interests from the beginning. This enables finding more stable solutions (agreements), which make participating agents less prone to deviating from the socially-optimal solution to favor their privately-optimal solution.

Particularly, this work is, to the best of our knowledge, the first one that deals with the frequency assignment problem by using non-linear negotiation techniques, which have been extensively studied by the authors [18,19]. This work contributes to achieving this objective in the following ways:

- We present the problem of frequency assignment in WSSNs.
- We model the problem of frequency assignment in WSSNs by means of the mathematical problem of graph coloring.
- We present a utility model for frequency assignment that takes into account the influence of interferences in terms of the signal-to-noise ratio in a receiver at a given time.
- We solve the frequency assignment problem in WSSNs with different negotiation techniques and, also, for comparison purposes, with other techniques: a random assignment, an optimization technique, and a heuristic technique based on LCCS.

The paper is structured as follows. In Section 2, we model the problem as a graph, and we also model the propagation, interferences and utility of the solutions. In Section 3, we describe in detail the automated negotiation techniques proposed to solve the frequency assignment problem. Finally, in Section 4, we describe the scenarios under study and show the results, while in Section 5 we conclude and discuss the paper.

## 2. Problem Modeling

### 2.1. WSSN Architecture

The architecture chosen for the WSSN is a single-tier clustered-based architecture [1] where IP-based cameras (sensors) are wirelessly connected to a central clusterhead, which is also in charge of processing the multimedia data (audio, video and possibly other scalar sensor data) received from the cameras. To cope with the high bandwidth requirements of multimedia transmission and the described architecture, we have resorted to IEEE 802.11n (Wi-Fi) technology working in the 2.4-GHz band. In this architecture, the clusterhead will be a device connected to (or integrated in) the Wi-Fi access point. As this architecture uses existing Wi-Fi networks, we have considered that, in addition to the cameras, there are other wireless devices in the Wi-Fi network, which are connected to the Internet through their associated access point.

**Figure 1:**
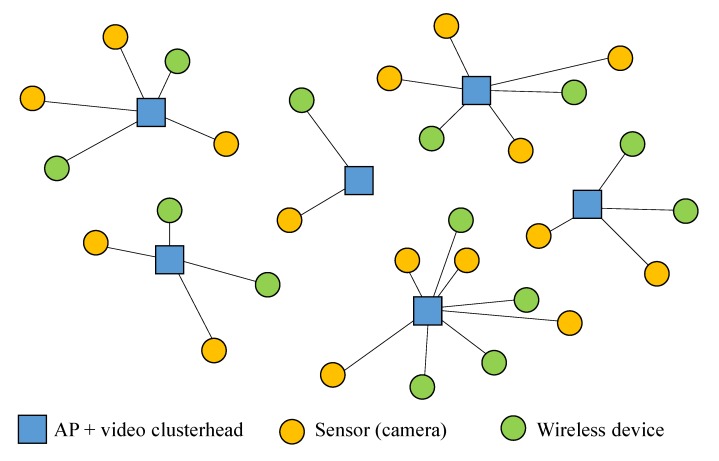
Wireless surveillance sensor network (WSSN) architecture.

Figure 1 shows a graphical view of the architecture. Although there are other technologies for sensor networks, like ZigBee or IEEE 802.15.4, these technologies have a clear shortcoming that impedes their use in WSSNs, as their transmission data rate is limited to 250 kbps, which makes real-time video transmission almost impossible [20] if we want to meet the quality standards needed for surveillance. Wi-Fi, on the contrary, provides the necessary bandwidth to transmit high-quality video in sensor networks [21,22,23].

Obviously, the main drawback of using Wi-Fi technology instead of ZigBee is that it is a less energy-efficient solution. For example, in [22], the authors propose a video sensor using Wi-Fi and estimate that Wi-Fi networking accounts for about a third of the total energy consumed by cameras. However, there are already studies that deal with this issue. In [24,25], the power consumption of video sensors using Wi-Fi is analyzed, while in [20], a dual radio proposal is discussed, where sensors use ZigBee when they transmit small amounts of data and Wi-Fi when they must transmit large amounts of data to the home router in order to reach a remote node connected to the Internet. In [26], the authors describe an energy-harvesting technique to use Wi-Fi for sensor networks.

### 2.2. Abstract Modeling Based on Graph Coloring

Graphs are one of the most commonly-used tools for modeling the frequency assignment problems, because of the relation of this problem to the graph coloring problem, which has been widely studied by the mathematical community [27].

In graph coloring, an abstract graph is considered, defined by a set of vertices along with some edges connecting them, and the objective is to assign one color to each vertex, in such a manner that the minimum possible number of colors should be used, while avoiding monochromatic edges.

In the commonly-used model, graph nodes represent elements that should be assigned a frequency, while edges represent element pairs that should not be assigned the same frequency. This way, colors act as frequencies, and hetero-chromatic edges guarantee element pairs with different frequencies.

Although widely used, Tragos *et al.* [28] conclude that the model is not accurate enough, because it does not reflect all of the information. For instance, the authors suggest that the information regarding adjacent channel interferences should be incorporated into the graph. In this work, we do so and use a graph showing greater fidelity to the problem modeled.

We can distinguish three types of vertices and two types of edges as graph elements. Regarding vertices, we consider access points (AP), sensors or cameras (C) and other wireless devices (D). Regarding edges, on the one hand, every wireless device (C or D) will be associated with its closest AP, trying to reflect that it will use the channel assigned to its closest AP. On the other hand, we will also link some node pairs when the distance between them is below the corresponding interference radius *R* (to reflect interferences): AP-AP pairs will be linked provided that the distance condition is met, AP-C (AP-D) pairs only when the camera (device) is not associated with that AP and C-C (D-D) pairs only if both cameras (devices) are associated with different APs, as the communications among the elements connected to the same AP are coordinated.

Furthermore, to model the interference power between two elements, we weigh each edge based on three factors. First, we consider a weight for each color pair ij, which can be understood as the interference between color *i* and color *j*. It is worth noting that the usual coloring problem only takes into account the particular case of interferences between vertices of the same color, while our extension of the problem allows considering also interferences between adjacent colors or colors in a certain distance range.

Second, we weigh the previous weights, by introducing the distance between edges’ endpoints. This way, the weight assigned to a colored edge ij will be different depending on how far apart its endpoints are. This represents another extension to the usual coloring problem, because now vertices have also certain positions, and this means that our graph is no longer abstract, but geometrical.

Third, we include the effect of the amount of data into the weights, including a factor that accounts for the fact that a higher bandwidth data flow will occupy the wireless channel a higher fraction of the time.

### 2.3. Effect of Propagation on Coverage

In order to evaluate a certain coloring for the proposed scenario, the effect of both the propagation and the interferences over wireless signals must be incorporated. Regarding the propagation model, in [29], it is defined that radio signal power losses, expressed in dB and considering that antennas are close to the ground (between 1 m and 2.5 m), can be obtained according to the following equation:

(1) $$P_{loss}=40\log_{10}d+20\log_{10}f-20\log_{10}\left(h_{t}h_{r}\right)$$

where *d* is the distance, in meters, *f* is the signal frequency, in GHz, and $h_{t}$ ($h_{r}$) is the transmission (reception) antenna height, also in meters. Equation (1) can be simplified for its use in the 2.4-GHz frequency band, and the resulting expression is as follows:

(2) $$P_{loss}=7.6+40\log_{10}d-20\log_{10}\left(h_{t}h_{r}\right)$$

A particularly interesting case of this propagation model is when a signal with a power equal to the receiver sensitivity is received, that is:

(3) $$P_{t}+G_{t}+G_{r}-L-P_{loss}=S$$

where $P_{t}$ stands for the transmission power (in dBm), $G_{t}$ ($G_{r}$) stands for the transmission (reception) antenna gain (in dB), *L* stands for losses due to walls, windows and other obstacles in the propagation (in dB), $P_{loss}$ stands for propagation losses (in dB) and *S* represents the receiver sensitivity (in dBm). Using Equation (2), we can determine the distance, such that signals can be perceived by the receiver. Such a distance (expressed in meters) can be obtained as:

(4) $$R=10^{\frac{P_{t}+G_{t}+G_{r}-L-S-7.6+20log_{10}\left(h_{t}h_{r}\right)}{40}}$$

The interest in computing *R* is that it allows one to compute the maximum distance between a pair of nodes for them to be connected in the interference graph.

### 2.4. Effect of Interferences

In order to be able to measure the quality of the received signal, we must take into account the interfering signals. By interfering signals, we mean those undesired signals received by different network elements that make it harder to receive the expected signal properly. In the context of this work, it is necessary to quantify the power of interfering signals both in wireless terminals (C and D) and APs. We have to take into consideration several issues for this. First, we have assumed that there are only interferences coming from other Wi-Fi devices, but not from any other wireless technologies that may be also using the 2.4-GHz frequency band. It would not be difficult to extend our interference model, but we have decided not to do so, because these interferences are unconnected to the problem that we are presenting here. Second, provided that distance also affects interference signals, those interferences coming from sources far enough from the reception point so that they fall below the receiver’s sensitivity have been dismissed, according to the interference graph presented in Section 2.2. Third, it is commonly accepted that data traffic in access networks is very asymmetric, *i.e.*, the downstream clearly dominates the upstream [30]. Indeed, this asymmetry has shaped the ADSL (Asymmetric Digital Subscriber Line) and cable design. For that reason, we have assumed that uplink traffic of wireless devices (D) is negligible in comparison with the traffic transmitted by APs, so they are not included in the graph. However, note that this assumption has only been made for wireless devices (D) different from cameras, as IP-based cameras’ (C) data traffic will be mainly upstream.

The quality of the received signal depends on all of the received interferences. Aside from distance (which affects signal losses; Equation (2)), interferences are also influenced by other factors: the activity index (Ψ) and the co-channel index (*δ*). The purpose of the activity index is to account for the fact that different volumes of data traffic will suppose different effects on interferences. In other words, higher bandwidth flows will generate more harmful interference signals, as they will occupy the spectrum for a higher ratio of time. Note that the interferences generated by APs are mainly due to traffic sent to the other wireless devices. Finally, the co-channel index (*δ*) is defined as the existing overlap between the IEEE 802.11 2.4-GHz frequency band. To model this effect, we have used the values obtained for this index in [31], where the authors provide a matrix where each value (i,j) represents the interference, as seen in channel *i*, motivated by the transmission on channel *j*. As a summary, if a node operating in channel *i* is interfered by a node operating in channel *j*, that interference power can be expressed as:

(5) $$I=P_{t}+G_{t}+G_{r}-L-P_{loss}+\delta \left(i,j\right)+\Psi$$

where every value is expressed in dB. Once there is a model for interfering signals, the signal-to-noise ratio for terminal *i* ($SINR_{i}$) can be computed as the ratio between the received signal and the sum of the received interferences, *i.e.*,

(6) $$SINR_{i}=\frac{P}{\sum_{j=1}^{M}I_{j}}$$

being *P* the power of the desired video signal and *M* the number of interference signals ($I_{j}$) that are received.

Note that each AP will have a SINR value for every terminal that is associated with it. In that case, we will assume that its SINR will be the minimum of all of them, which is in fact the worst case.

### 2.5. Utility of the Solutions

**Figure 2:**
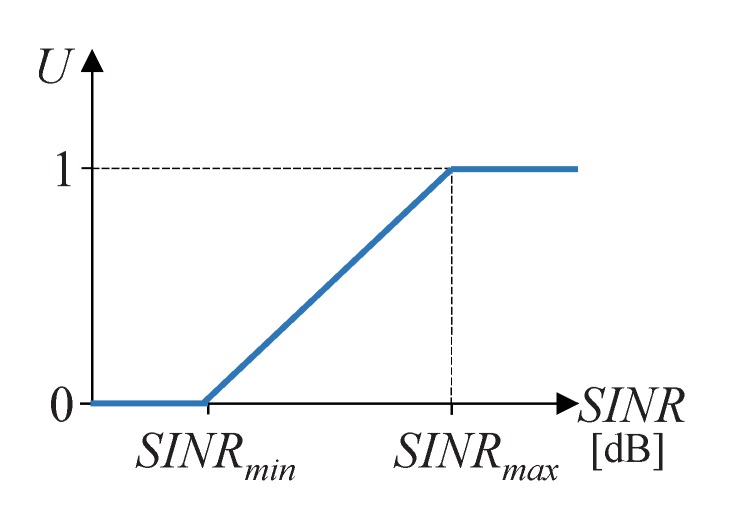
Relation between utility and SINR.

To quantify the goodness of the different network colorings, we have used the concept of utility, which is closely related to the perceived throughput and SINR. According to [8], in a wireless network, the throughput equals a maximum value when the SINR is over a certain value $SINR_{max}$ and monotonically decreases with the reduction of SINR until an insufficient value of SINR, called $SINR_{min}$, is reached, when the throughput falls to zero. We can consider the utility seen by node *i* ($U_{i}$) as a normalized throughput, so it can be defined as a value ranging from zero to one, with zero corresponding to the situations when there is a very low-quality reception and the devices cannot keep connected (throughput equals zero) and one corresponding to the case when the signal quality is excellent (throughput equals its maximum value). Figure 2 shows the relation between utility and SINR, expressed in dB.

Threshold values for SINR, which in turn define maximum and minimum utility values, have been defined from the values presented in [32]: $SINR_{min}=$ 10 dB and $SINR_{max}=$ 40 dB.

Finally, the utility value for a specific network coloring is computed as the sum of the utility values for all nodes in the network, that is:

(7) $$U=\sum_{\forall i}U_{i}$$

In a wireless sensor network, it is important to consider sensor energy consumption issues, as they will have a clear impact on sensor lifetime. However, a surveillance camera will consume the same energy independent of the frequency channel on which it is operating. For that reason, the different SINR values that the different strategies provide come from different values in the interferences (denominator in the SINR equation), not by different values in the signal (numerator in the SINR equation).

## 3. Automated Negotiation Techniques for Channel Selection

In this work, we propose to tackle the frequency assignment problem in WSSNs using automated negotiation techniques. Automated negotiation is a quite wide [33] field, but most authors agree that a negotiation problem can be characterized by a negotiation domain (who negotiates and what to negotiate about), an interaction protocol (which rules govern the negotiation process) and a set of decision mechanisms or strategies that guide the negotiating agents through every phase of the interaction protocol [34]. In the following, we define our particular negotiation problem along these three dimensions.

### 3.1. Negotiation Domain

For the scope of this work, we assume a multi-attribute negotiation domain, where a deal or solution to the problem is defined as the set of attributes (issues), and each one of them can be in a certain range. In our case, for a channel assignment problem with $n_{AP}$ access points, a solution or deal *S* can be expressed as $S=\{s_{i}|i\in 1,...,n_{AP}\}$, where $s_{i}\in \left\{1,\ldots ,11\right\}$ represents the assignation of a Wi-Fi channel to the *i*-th access point. Although there are 13 channels in the 2.4-GHz frequency band, they are not used everywhere in the world. For instance, only the first 11 channels are used in North America. In this work, we will use only those 11 channels, as the co-channel interference computation, referred in the previous section, has been done after the work in [31], where only 11 channels are used.

In this work, we assume that there are a number *p* of network providers offering surveillance services; thus, APs can belong to any of the providers $P_{i}$. Each provider only has control over the frequency assignment for its own access points. According to this situation, $P=\{p_{i}\}|i=1,...,p$ will be the set of agents that will negotiate the frequency assignment.

**Figure 3:**
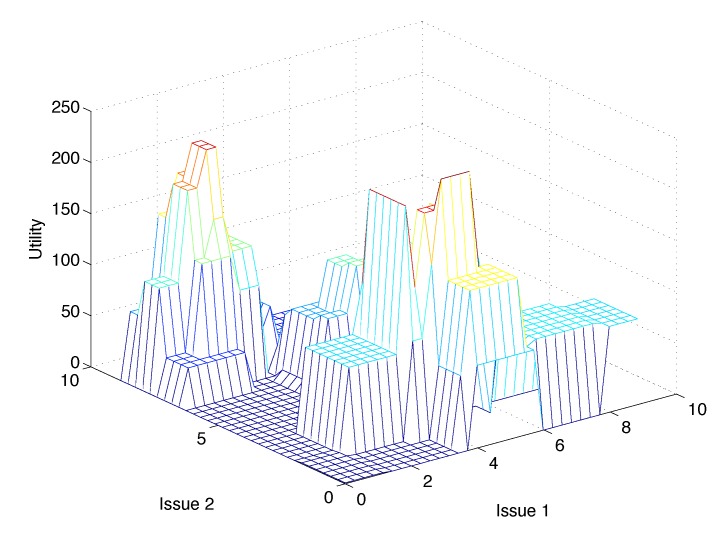
Utility function with multiple local maxima.

Finally, each one of these agents will compute its utility for a certain deal or solution according to the model described in the previous section, but taking into account only the interference affecting its own access points. Our hypothesis is that the problem settings (high cardinality of the solution space and attribute interdependence) will make the utility functions highly complex, with multiple local minima and maxima. Figure 3 shows an example of such a complex utility function for two issues. Unfortunately, the dimensionality and cardinality of the utility spaces derived from the setting of this problem will prevent us from providing a graphical representation of the actual utility functions (or even sampling them). Nevertheless, in Section 4.3, we will present some results that support this assumption.

### 3.2. Interaction Protocol

There are many interaction protocols for negotiations, from the classical alternating offers model [35] to auction-based protocols [36]. Accepting the hypothesis that the negotiation scenarios coming from the channel assignment problem in WSSNs will be highly nonlinear and according to the discussion in [37], we have chosen a simple text mediation protocol [38]. In its simplest version, the negotiation protocol will be as follows:

1. It starts with a randomly-generated candidate contract ($S_{0}^{c}$). In our case, this means assigning a random channel to each access point.
2. In each iteration *t*, the mediator proposes a contract $S_{t}^{c}$ to the rest of agents.
3. Each agent either accepts or rejects the contract proposed by the mediator.
4. The mediator generates a new contract $S_{t+1}^{c}$ from the previous contracts and from the votes received from the agents, and the process moves to Step 2.

This process goes on until either a maximum number of iterations is reached or another stop condition is met. The protocol, as defined, is rather generic and must be completed with the definition of the decision mechanism or strategies to be used by the negotiating agents and the mediator.

### 3.3. Decision Mechanisms

For the mediator, we have implemented a single-text mediation mechanism [38] for the generation of new contracts, which works as shown in Algorithm 1:

- If at time *t* all agents have accepted the presented contract $S_{t}^{c}$, this contract will be used as the base contract $S^{b}$ to generate the next contract $S_{t+1}^{c}$ (1). Otherwise, the last mutually-accepted contract will be used.
- To generate the next candidate contract $S_{t+1}^{c}$, the mediator takes the base contract $S_{b}$ and mutates one of its issues randomly (2). In our case of study, this would correspond to choosing a random access point and selecting a new random channel for it.
- After a fixed number of iterations, the mediator advertises the final contract, which will be the last mutually-accepted contract (3).

For the agents, we have considered two different mechanisms to vote about the candidate contracts $S^{c}$:

- Hill-climber (HC): In this case, the agent behaves as a greedy utility maximizer (see Algorithm 2). The agent will only accept a contract when it has at least the same utility for her or him as the previous mutually-accepted contract (2). If there is no previous mutually-accepted contract (1), the agent will accept the presented one (this effectively makes the first contract generated by the mediator be the first mutually-accepted contract).
- Annealer (simulated annealing (SA)): In this case, we use a widespread nonlinear optimization technique called simulated annealing [38,39]. When a contract yields a utility loss against the previous mutually-accepted contract, there will be a probability for the agent to accept it nonetheless. As shown in Algorithm 3, this probability $P_{a}$ depends on the utility loss associated with the new contract Δu and also depends on a parameter known as annealing temperature *τ*, so that $P_{a}=e^{\frac{-\Delta u}{\tau }}$ (2). Annealing temperature begins at an initial value and linearly decreases to zero throughout the successive iterations of the protocol (1).

**Algorithm 1:** Single-text mediation algorithm.
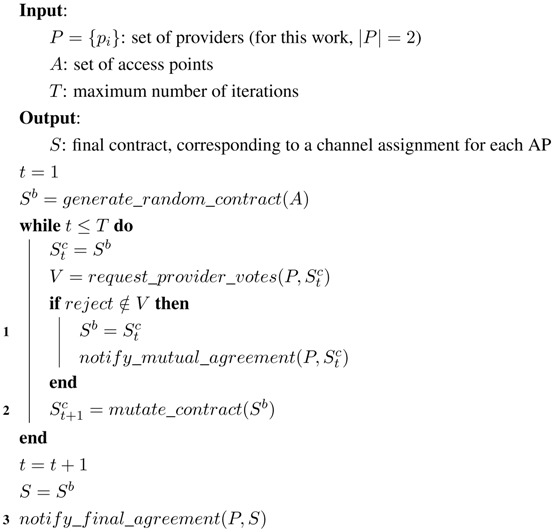

**Algorithm 2:** Hill-climber voting algorithm.
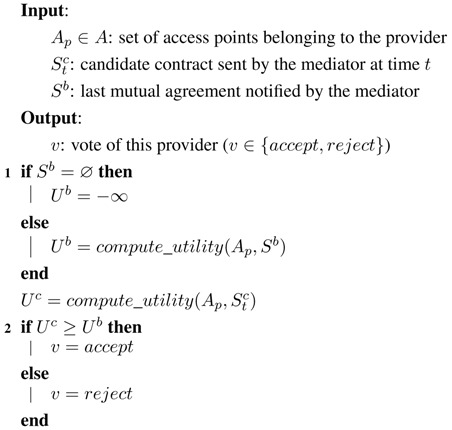

**Algorithm 3:** Annealer voting algorithm.
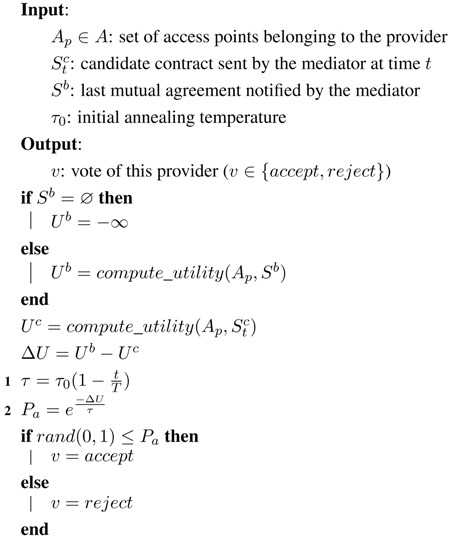


The choice of these two mechanisms is not arbitrary. Simulated annealing techniques have yielded very satisfactory results in negotiation for nonlinear utility spaces [40] and are the basis for several of our previous works [18,19]. Furthermore, as discussed in [38], the comparison between hill-climbers and annealers allows one to assess whether the scenario under consideration is a highly complex one, since in such scenarios, greedy optimizers tend to get stuck in local optima, while the simulated annealing optimizer tends to escape from them.

For a complexity analysis, we are using heuristics that are stopped when reaching a certain number of iterations (see Section 4.3). For the complexity of each iteration, we first have to compute the utility of the corresponding coloring (see Section 2.5), which requires checking the neighbors for every node and, hence, lies in $O(n^{2})$ for *n* the number of nodes in the network. After that, the negotiation steps detailed in this section arise, with a constant complexity in every iteration. Therefore, the complexity of our results is in $O(n^{2})$.

## 4. Experimental Evaluation

### 4.1. Considered Scenarios

In this paper, we make the common assumption that sensor nodes (IP-based cameras) are static elements. As in our problem there is not any element that evolves with time, we deal with the problem of evaluating the performance of a particular frequency assignment strategy by means of the computation described in Section 2. Please, note that the frequency strategy evolves with time, but it is computed in an offline manner.

We have taken into consideration three types of WSSN scenarios. In the first case, we have considered 50 APs and 350 (50 × 7) cameras; in the second one, 50 APs and 500 (50 × 10) cameras; and in the third one, 100 APs and 500 (100 × 5) cameras. The position where APs and wireless terminals are located in a plane is random, considering that a wireless terminal is associated with its closest AP. If a sensor (camera) is not in the coverage area of any AP (given by the sensitivity of its receptor, as explained in Section 2.3), we have removed that sensor from the problem. Additionally, if an AP has no associated sensors, it is also removed from the problem. Obviously, as the density of nodes increases, the number of unconnected nodes decreases. For each type of scenario and due to their randomness, we have generated three specific scenarios, so we have studied nine scenarios. Moreover, given the non-deterministic nature of the different algorithms under study, we have run each algorithm ten times in each scenario. Table 1 summarizes the scenarios under study, where we show the number of nodes (APs and sensors) initially deployed and the number of nodes that remain after removing the unconnected nodes (*ν*). Moreover, for the sake of quantifying the density of each scenario, we also show the mean number of interference signals that each node in the network receives ($\bar{I}$).

**Table 1:** Summary of scenarios.

Scenario	# APs	# Sensors	*ν*	$\bar{I}$
1	50	350	237	22.53
2	50	350	241	21.53
3	50	350	240	21.81
4	50	500	439	34.72
5	50	500	414	39.26
6	50	500	427	34.30
7	100	500	490	47.62
8	100	500	487	51.57
9	100	500	527	48.63

### 4.2. Analyzed Techniques

In addition to the negotiation techniques under study, presented in Section 3.3, we have included a comparison with three reference techniques:

- Random reference: As a first baseline, in this technique, each AP chooses a channel randomly to use with their clients.
- Sequential channel search (SCS): This is a more realistic baseline and inspired by LCCS [11]. In this algorithm, APs are activated in sequence, and each of them chooses the channel where it finds the lowest interferences from other active APs and their clients. If there are several least congested channels, it chooses one of them randomly.
- Augmented Lagrangian harmony search optimization (ALHSO): In addition to our negotiators based on simulated annealing, we wanted to have, as a reference, a nonlinear optimizer using complete information. We have chosen harmony search, which is an evolutionary optimization algorithm inspired by musical composition [41]. More concretely, we have used a public implementation that uses augmented Lagrange multipliers to deal with restrictions [42].

### 4.3. Results

The choice of the configuration parameters for the studied scenarios has been driven by considering typical or reasonable parameters from a realistic point of view, as summarized in Table 2. With these values, the coverage area is R=40.3 m.

**Figure 4:**
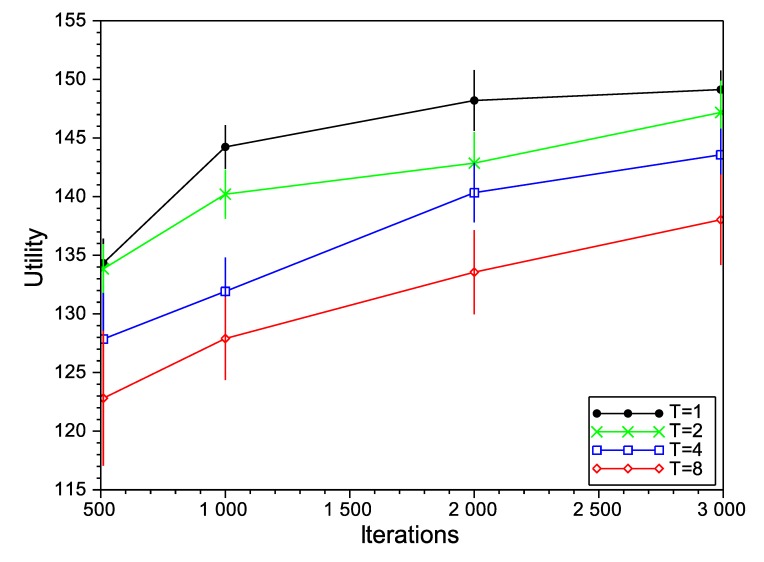
Simulated annealing (SA) evaluation in Scenario 3.

**Figure 5:**
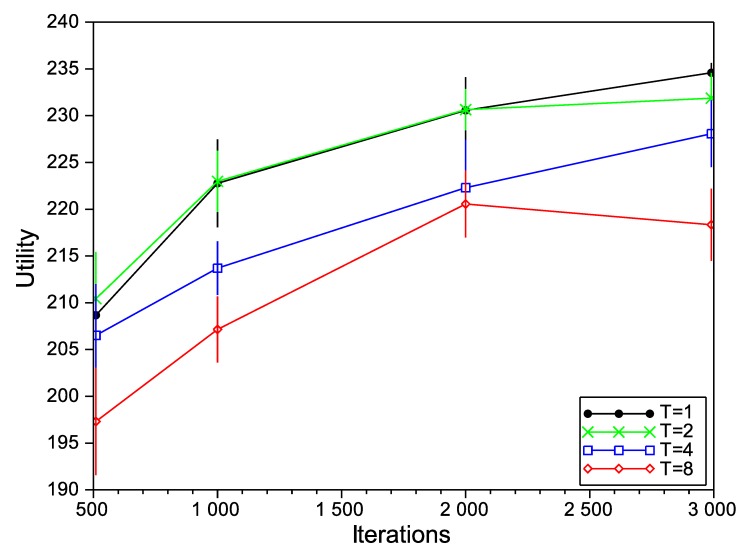
SA evaluation in Scenario 6.

**Figure 6:**
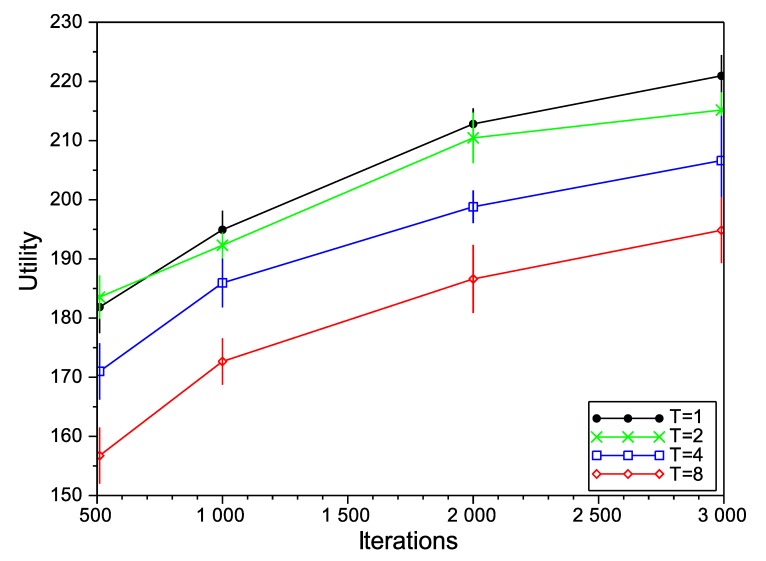
SA evaluation in Scenario 9.

Firstly, we study the performance of the SA algorithm with different values for its configuration parameters: initial temperature (*T*) and the number of iterations. In general, a higher initial temperature increases the chances for escaping from local maxima, although an excessive temperature can drive the optimizer to escape from the global maximum. For that reason, setting the initial temperature is usually a delicate matter and depends on the concrete application. For the case of the number of iterations, and as a general rule, a higher number of iterations provides more opportunities to improve the solution, but it also increases the time that the algorithm is operating with high temperatures and, so, the risk of escaping from the global maximum (in addition to the increased computation cost, of course). In Figure 4, Figure 5 and Figure 6, we show the variation of the utility and the confidence intervals at 95% of the final negotiation outcome for different values of temperature and the number of iterations for Scenarios 3, 6 and 9 (we omit the rest of the scenarios, as the results obtained are very similar). In those figures, we notice that, for the number of iterations, the best choice is to choose the highest number as possible to obtain higher utilities. This effect occurs in all of the studied scenarios (not only the ones depicted in the figures), with the exception of one of them (Scenario 6 in Figure 5 with T=8), where the utility function decreases from 220.56 to 218.34 for 2000 and 3000 iterations, respectively. Regarding the initial temperature *T*, the best choice is T=1. From this point on, the SA algorithm will be executed with T=1 and 3000 iterations.

**Table 2:** Summary of the parameters (C, camera).

Parameter	Value
$P_{t}$	30 mW
$G_{t}$	0 dB
$G_{r}$	0 dB
*L*	40 dB
*S*	-90 dBm
$h_{t}$	1.5 m
$h_{r}$	1.5 m
Ψ (APs)	0.5
Ψ (C)	0.2

**Figure 7:**
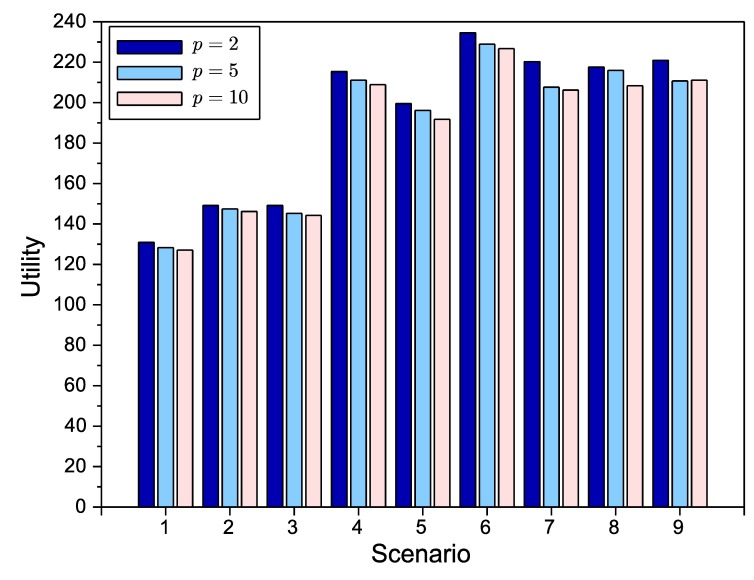
SA evaluation with different numbers of network providers (*p*).

Once the SA algorithm has been tuned, the next experiments are focused on determining the impact of having a different number of agents or network providers (*p*) in the negotiation. In Figure 7, we have evaluated all of the scenarios under study with p∈[2,5,10]. From the figure, we can conclude that, as expected, increasing the number of providers *p* yields a progressive diminution of the global utility, since trade-off solutions involving access points in different providers will be more likely to be rejected. However, this reduction is moderate enough. From this point on, all of the results belong to the case p=2. The reason for this choice is two-fold. First, we think it is reasonable to consider a small number of providers offering surveillance services. Second, there are more works in complex bilateral negotiations than for the multilateral case (three or more agents).

Next, we compare the SA algorithm with the rest of the proposals: random channel assignment, SCS, HC and ALHSO. In Table 3, Table 4 and Table 5, we show the average (avg), standard deviation (SD) and confidence intervals at 95% (CI) of the utility function for 10 executions of the algorithm in each of the proposed scenarios. Furthermore, we highlight in bold the best solution. As can be observed, regarding the mean utility, the SA algorithm is the best solution for all of the scenarios, except for Scenario 8, where ALHSO shows the best performance. The second best performance is obtained by ALHSO, followed, by far, by SCS. As could be expected, the worst performance is obtained by the random channel assignment, which obtains very low utility values. In the case of HC (which can be seen as an SA optimizer with T=0), we can note that it is able to obtain good results for the simplest scenarios (Table 3), but its performance decreases in more complex scenarios (Table 3, Table 4 and Table 5), due to its tendency of getting stuck in local maxima.

**Table 3:** Utility in scenarios with 50 APs and 350 sensors. SCS, sequential channel search; ALHSO, augmented Lagrangian harmony search optimization; HC, hill-climber.

	Scenario 1			Scenario 2			Scenario 3		
	avg	SD	CI	avg	SD	CI	avg	SD	CI
Random	59.36	9.61	6.87	73.74	10.68	7.64	65.81	13.76	9.84
SCS	106.74	4.30	3.08	121.20	7.18	5.14	122.78	4.46	3.19
ALHSO	121.64	4.17	2.98	141.09	3.56	2.55	132.92	3.95	2.83
HC	123.30	3.50	2.50	140.37	4.93	3.53	138.02	5.31	3.80
SA	**130.89**	2.65	1.90	**149.16**	1.65	1.18	**149.13**	2.27	1.62

**Table 4:** Utility in scenarios with 50 APs and 500 sensors.

	Scenario 4			Scenario 5			Scenario 6		
	avg	SD	CI	avg	SD	CI	avg	SD	CI
Random	92.96	6.59	4.71	78.05	16.95	12.13	105.08	13.70	9.80
SCS	165.60	9.00	6.44	151.74	10.24	7.33	181.27	9.14	6.54
ALHSO	203.62	5.76	4.12	184.01	7.46	5.34	221.96	5.37	3.84
HC	196.82	9.94	7.11	179.11	11.06	7.91	211.76	11.05	7.90
SA	**215.37**	2.75	1.97	**199.52**	2.98	2.13	**234.59**	1.46	1.04

**Table 5:** Utility in scenarios with 100 APs and 500 sensors.

	Scenario 7			Scenario 8			Scenario 9		
	avg	SD	CI	avg	SD	CI	avg	SD	CI
Random	90.13	9.14	6.54	95.73	15.87	11.35	88.79	11.64	8.33
SCS	164.94	9.34	6.68	168.28	13.09	9.36	172.70	11.53	8.25
ALHSO	216.36	6.40	4.58	**223.02**	5.03	3.60	217.53	4.86	3.48
HC	196.33	8.07	5.77	199.51	8.74	6.25	199.65	7.73	5.53
SA	**220.29**	5.17	3.70	217.60	5.24	3.75	**220.94**	4.92	3.52

Regarding the standard deviation of the solutions, the algorithm that shows the lowest deviation is SA in almost every studied case (except in the most complex Scenarios 8 and 9, where SA is very similar to ALHSO), followed by ALHSO, SCS and random. As a conclusion, we can state that outperforming an optimization algorithm like ALHSO with negotiation techniques like SA shows that the use of negotiation techniques is advisable for the problem under study. Since the SA algorithm requires, as seen previously, the configuration of two parameters (initial temperature and the number of iterations), we finally include a comparison of all of the algorithms in a relative manner, with the purpose of analyzing the behavior of SA if it is not configured properly.

**Figure 8:**
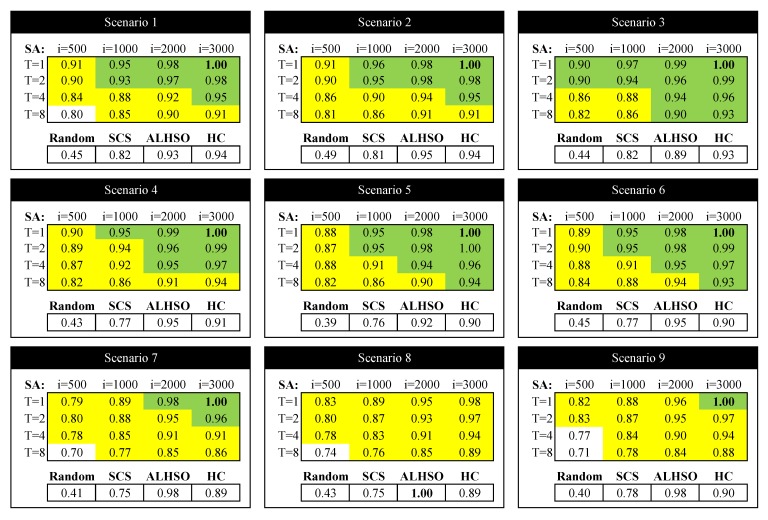
Results relatives to the maximum.

Figure 8 shows the mean value of the utility functions for the different scenarios and relative to their maximum value (in bold). To make the interpretation easier, in the table devoted to the results of the SA algorithm, we have highlighted in green those results that outperform or equal ALHSO (they also outperform SCS and random, as they are less restrictive). Moreover, we have highlighted in yellow the results where SA outperforms SCS. As a conclusion, for temperatures close to T=1 and 1000 or more iterations, SA outperforms ALHSO. Moreover, we notice that, for almost all cases (except for very high temperatures and very few iterations), SA outperforms SCS. In the figure, we also show the results for HC (equivalent to SA with T=0) with 3000 iterations, and we observe that it has a worse performance than SA for the same number of iterations.

As a conclusion, we can recommend the use of negotiation techniques based on SA, as it is able to obtain better results than SCS in almost all cases, and it is not difficult to find configuration parameters that make SA even better than a nonlinear optimizer like ALHSO.

## 5. Discussion and Conclusions

Frequency assignment in Wi-Fi-based WSSNs is a topic that has not yet attracted the necessary attention from the research community. This scarcity of related works is probably due to the complexity of the problem, which is NP-hard [43]. More specifically, we have modeled the problem by using graphs, so the channel assignment is equivalent to coloring the edges of a graph. We have included the effect of having partially-overlapped channels (as is the case in IEEE 802.11n) by using a co-channel interference matrix. In fact, in [43], the authors present a summary of techniques useful for assigning frequencies in Wi-Fi, where it can be concluded that there are not too many proposals. Regarding other research works related to our proposal, it is important to cite [44,45,46], this last work being the one that is probably more related to our proposal, as it is inspired by the graph coloring problem and uses a measurement to quantify the performance of the solutions, which is similar to the utility that we use in negotiation. However, none of these works [44,45,46] is focused on WSSNs and the use of negotiation techniques. A proposal that deals with the coordination of Wi-Fi access points to assign frequencies without the use of graphs can be found in [47].

From a practical point of view, and to the best of our knowledge, probably the only technique that is actually used is the one called LCCS [11]. This technique is quite simple: if an access point detects too many interferences in the channel it is using, it moves to the least congested channel that can be found at that moment. In this paper, we show that there is still much more room for improvement, so it is possible to reach solutions that clearly improve the spectrum usage of WSSN. More concretely, we have used a bilateral negotiation protocol with a mediator, where negotiating agents (two access providers, each of them controlling a fraction of the access points in the scenario) negotiate with a strategy based on either HC or SA. We show that automated negotiation techniques are able to obtain those improvements for both the mean and standard deviation of the utility. Moreover, we have compared those results with those obtained by the optimization technique called ALHSO, achieving fairly similar results. However, negotiation techniques are simpler and less time consuming than ALHSO. As a general conclusion, we can state that negotiation techniques are very useful to solve the problem of frequency assignment in WSSN.

Finally, although our experiments have yielded satisfactory results, the work presented here is not exempt from limitations. The use of negotiation techniques such as the ones described here imposes a communication and computational overhead over the negotiating elements. Taking this into account in our approach, only the APs (which in this setting are assumed not to be battery powered) participate in the negotiation. Moreover, in this work, we have assumed the bandwidth cost of negotiation messages as negligible when compared to the high volume of data exchanged in video surveillance networks. For settings with lower application bandwidth consumption, mechanisms that minimize the number of exchanged messages (e.g., one-shot combinatorial auctions) may be more adequate.

This works opens a variety of avenues for further research. As discussed in the previous sections, a range of bilateral and multilateral negotiation protocols and agent decision mechanisms can be studied. We are interested in determining which features of different WSSN scenarios may help us determine the more suitable negotiation approach to be used in a specific situation, in a similar way as we did in [48]. Furthermore, we are working in more complex mediation approaches, which may improve the efficiency of the negotiations while enforcing different social welfare notions in the outcomes. Finally, we are interested in fully-distributed negotiations, where the need for mediation can be substituted by a form of distributed social choice.
